# How Impulsiveness Influences Obesity: The Mediating Effect of Resting-State Brain Activity in the dlPFC

**DOI:** 10.3389/fpsyt.2022.873953

**Published:** 2022-05-10

**Authors:** Xiao-Dong Han, Hong-Wei Zhang, Ting Xu, Lin Liu, Hui-Ting Cai, Zi-Qi Liu, Qing Li, Hui Zheng, Tao Xu, Ti-Fei Yuan

**Affiliations:** ^1^Department of Metabolic and Bariatric Surgery, Shanghai Jiao Tong University Affiliated Sixth People's Hospital, Shanghai, China; ^2^Shanghai Key Laboratory of Psychotic Disorders, Shanghai Mental Health Center, Shanghai Jiao Tong University School of Medicine, Shanghai, China; ^3^MR Collaborations, Siemens Healthcare Ltd., Shanghai, China; ^4^Department of Anaesthesiology, Affiliated Shanghai Sixth People's Hospital, Shanghai Jiao Tong University, Shanghai, China; ^5^Department of Anaesthesiology, Tongzhou People's Hospital, Nantong, China; ^6^Co-innovation Center of Neuroregeneration, Nantong University, Nantong, China; ^7^Translational Research Institute of Brain and Brain-Like Intelligence, Shanghai Fourth People's Hospital Affiliated to Tongji University School of Medicine, Shanghai, China

**Keywords:** obesity, impulsiveness, resting-state fMRI, mediation effect, dlPFC

## Abstract

Impulsiveness is a stable personal characteristic that contributes to obesity and may interact with it. Specifically, obesity is caused by unrestrained impulse eating that is not consciously controlled and leads to a hormonal imbalance that also can impair impulse control. However, the mechanism of this relationship is unclear. In our study, 35 obese individuals (body mass index, BMI > 28) were recruited and matched with 31 healthy controls (BMI < 24) in age and education level. All the participants underwent a resting-state fMRI and completed the Barratt Impulsiveness Scale-11. The results showed that patients with obesity had a significantly lower fractional amplitude of low-frequency fluctuations (fALFF) in the bilateral dorsolateral prefrontal cortex (dlPFC) and higher fALFF in the left fusiform cortex. In addition, non-planning impulsiveness was positively correlated with BMI. Importantly, we found that the right dlPFC completely mediated the relationship between non-planning impulsiveness and BMI. Our findings suggest that impulsivity is statistically more likely to precede obesity than to precede impulsivity and contributes to obesity by downregulating spontaneous activity in the dlPFC. This suggests that the dlPFC, which is associated with executive control, may be able a potential target for treating obesity.

## Introduction

Currently, humans can rapidly obtain food and therefore require more effort to control their appetites and eat healthily. The personality trait of impulsiveness is a risk factor for overeating and obesity (1). Beyond mere cosmetic concerns, obesity also poses a significant health risk for individuals, as it can lead to diabetes, cardiovascular disease, and cognitive impairment (2). The prevalence of obesity has increased globally since 1950, as 13% of adults (18 years or older) were obese in 2016 (3). Imbalances in energy consumption lead to obesity if more calories are consumed and/or less energy is expended (4). The link between impulsiveness and obesity is relatively straightforward: children that struggle with delayed gratification and poor impulse control are more likely to eat high-fat foods when they are available (5). One survey showed that patients with obesity are higher in impulsiveness (6), and impulsiveness can also significantly predict the development and treatment of obesity (7). Although there may be more complex associations between impulsiveness and obesity, excess body fat deposits (whether caused by diet or otherwise) have been shown to lead to low-grade inflammation and alter the production of hormones that affect neuronal structure and function related to motivation and impair executive control (8).

Obesity alters subcortical structures (i.e., the basal ganglia) that are related to motivation as well as cortical structures (i.e., the prefrontal cortex [PFC]) that are related to executive functions (9). In classic theory, the most powerful drive toward overeating is the motivation of hunger and cravings for specific food. Motivation-related subcortical structures include the nucleus accumbens (NAcc), which is related to reward, the amygdala, which is related to fear, and the striatum, which is related to habitual behaviors (10). A recent UK Biobank study showed that patients with obesity have lower gray matter volume of subcortical areas (the thalamus, caudate nucleus, putamen, globus pallidus, hippocampus, nucleus accumbens, and amygdala) and aberrant white matter microstructure (11). The behavioral outcome of the personality trait of impulsiveness seems to be a reduction in executive control that can lead to overeating. A brain perfusion scan study showed that in response to drinking glucose, obese adolescents show reduced cerebral perfusion in brain regions involved in executive function (the PFC) and increased perfusion in areas of the brain involved in homeostasis (the hypothalamus) (12). An magnetic resonance spectroscopy study demonstrated blunted brain energy consumption and impaired systematic glucose uptake in obese participants compared with normal-weight participants, indicating neuroenergetic dysregulation in the insula (13). However, it remains unclear which specific brain regions play an important role in impulsiveness and obesity.

The dorsolateral prefrontal cortex (dlPFC) is a key region of executive control, which is also involved in impulsive decision-making (14) and is correlated with impulsiveness (15). The resting-state functional magnetic resonance imaging (rs-fMRI) index seems to reflect personality (16). ALFF is a common resting-state fMRI index that indicates improvement in brain functions and provides a potential target for clinical settings, such as neuromodulation. Low frequency (typically 0.01–0.08 Hz) fluctuation is a stable indicator of spontaneous neural activity (17) that has been used to detect changes in brain state in various diseases, including Alzheimer's disease (18), schizophrenia (19), and obesity (20). In 2018, one study found that the ALFF of the hippocampus in 30 obese patients decreased significantly after bariatric surgery and was negatively correlated with postoperative leptin and anxiety (21). In addition, this study found that 1 month postoperatively, the ALFF was changed and that there was not a group difference between patients with obesity and healthy controls (HCs). This may be because the ALFF is sensitive to physiological noise and not very robust. Previous studies have proposed utilizing the fractional ALFF (fALFF) method, that is, the ratio of the power spectrum of the low-frequency range (0.01–0.08 Hz) to the power spectrum of the entire frequency range, which more accurately reflects the intensity of brain activity (22).

Recent evidence has suggested that patients with obesity show increased impulsiveness and that increased impulsiveness also predicts a higher body mass index (BMI) (23–25). It is also known that executive function regions such as the dlPFC play an important role in moderating impulsiveness (26). However, it is extremely challenging to determine exactly how impulsiveness interacts with obesity, especially since the brain modifies this relationship. Evidence on the mediating role of resting-state brain activity in the relationship between impulsiveness and obesity is lacking. In this study, we performed a resting-state analysis to map fALFF patterns and correlated those patterns with BMI. The results of this investigation could be a major step toward understanding the precise relationship between impulsiveness and obesity. We hypothesized that (1) altered spontaneous activity would be observed in the prefrontal region and that (2) this activity would mediate impulsiveness and BMI. This study was preregistered in the open science framework (https://osf.io/rkwn8).

## Methods

### Participants

From September 2020 to December 2021, we recruited 37 obese subjects (BMI > 28, age from 19.95 to 49 years old, 7 male) through the Weight Loss Metabolism Clinic in China and 33 healthy volunteer (BMI < 24, age from 21.59 to 56.3 years old, 9 male) matched on demographic factors through local advertisements. According to the data processing flow, the magnetic resonance data of the last 35 obese patients and 31 healthy patients were included in the analysis. For the specific quality control of the subjects, see the data processing flow section. We found that these two groups have significant differences in BMI and non-planing impulsiveness. There was no group difference in Gender, Diabetic comorbidities, age, and years of education. For more information, see Table 1. All participants (1) were at least 18 years old; (2) could read and understand the description of each item in the questionnaire; and (3) signed written consent. There were exclusion criteria in both groups for people with neurologic diseases or other psychiatric disorders. (1) history of head trauma, (2) neurodevelopmental (intellectual disability), (3) neurocognitive (dementia) conditions, 4 addiction or substance use disorder. The ethics committee of Shanghai Sixth People's Hospital approved the study. All procedures were in accordance with the Declaration of Helsinki.

**Table 1:** Demographic information.

	**Obesity** **(Mean ±SD)**	**HC** **(Mean ±SD)**	**df**	***t/*χ^2^**	***p***	**Effect size**
Gender (M/F)	7/28	9/24	1	0.50	0.480	/
Diabetes	5/30	1/32	1	2.68	0.102	/
Obesity starts						
Child	20	/				
Adolescence	6	/				
Adult	6	/				
Fluctuation	2	/				
Other situation	1	/				
Age (years)	28.51 ± 5.89	30.49 ± 8.43	66	1.22	0.265	0.273
Education level	2.85 ± 0.66	3.22 ± 0.87	64^a^	1.88	0.058	0.476
BMI	37.6 ± 4.59	21.65 ± 2.06	66	−18.59	<0.001	−4.442
Nonplan imp	42.06 ± 20.21	32.11 ± 15.54	64^a^	−2.16	0.029	−0.55
Motor imp	39.49 ± 14.03	43.52 ± 9.94	64^a^	1.40	0.185	0.33
Cog imp	42.5 ± 16.16	46.09 ± 10.83	64^a^	1.11	0.296	0.26
Average imp	41.35 ± 12.90	40.57 ± 6.09	64^a^	−0.31	0.758	−0.076

*HC, healthy Control; SD, Standard deviation; df degree of freedom; p p value; Effect Size for t test is Choen's d; Gender was counted in number, M, Male, F, Female; Education level, The number of years of education has been coded, 6–9 years as 1, 9–12 years are coded as 2, 12–16 years are coded as 3, and more than 16 years are code as 4; non-planning impulsiveness (Nonplan imp), motor impulsiveness (Motor imp), cognitive impulsiveness (Cog imp), and average score of three subscale (Average imp). ^a^Two patients in HC group had failure to finish the questionnaire, so the information is missed*.

### Procedure and Measurements

First, all participants were briefed on the goal of the experiment and completed the informed consent form. Then, basic demographic information (age, education level, and gender) was collected, and BMIs (equal to [weight in kilograms divided by height in meters]^2^) were calculated to determine the degree of obesity. Second, participants completed the Chinese version of the Barratt Impulsiveness Scale-11 (BIS-11) and underwent a 12-min MRI scan. Two participants in the HC group (2/33) didn't finish the main questionnaire, so remove from the following analysis.

The BIS-11 is a widely used 30-item scale that measures trait impulsiveness in adults (27). The Chinese version of the BIS-11 consists of three subscales: non-planning impulsiveness (non-plan imp), motor impulsiveness (motor imp), and cognitive impulsiveness (cog imp). Cognitive impulsiveness reflects impulsive thoughts and anticipation of the results of actions. Motor impulsiveness reflects the impulse to act. Non-planning impulsiveness reflects a lack of future planning (28). Each item was measured on a 5-point Likert score from 1 “never” to 5 “very often.” The score range for each subscale is from 10 to 50 points; the subscale scores and the total score are converted to a range of 0 to 100 points. That is, the score of each subscale = [(Sum of points of each item−10) ÷ 40] × 100,” and the average imp score = the sum of the three subscales/3. Higher scores indicate higher impulsiveness.

### MRI Acquisition and Processing

MRI data were acquired by a 3.0-Tesla Siemens Prisma scanner with a 64-channel head coil at the Shanghai Sixth People's Hospital (Shanghai, China). The fMRI images were acquired using an echo-planar imaging pulse sequence with the following parameters: total volume = 210, repetition time = 2,000 ms, echo time = 30 ms, flip angle = 90°, number of slices = 33, transverse orientation, field of view = 224 × 224 mm^2^, matrix size = 64 × 64, slice thickness = 3.5 mm. The structural images were acquired using a magnetization-prepared rapid gradient-echo sequence with the following parameters: repetition time = 2,000 ms, echo time 2.32 ms, inversion time = 900 ms, flip angle = 8°, number of slices = 208, field of view = 230 × 230 mm^2^, matrix size = 256 × 256, slice thickness = 0.9 mm.

The Data Processing Assistant for Resting-State fMRI v5.2 (http://rfmri.org/dparsf) was used to preprocess and analyse the MRI data. To prevent instability in the data, the first 10 volumes were discarded. After slice timing, reorientation, and realignment to the middle slice, T1 coregistration was applied. A skull-stripped T1 image was segmented using Diffeomorphic Anatomical Registration Through Exponentiated Lie (DARTEL) algebra. Covariances associated with nuisance variables were then regressed out. Motion was controlled with a 24-parameter Friston model (29). We excluded subjects if their head movements or rotation were >2.0° or 2.0 mm, resulting in the exclusion of two patients (2/37) and zero healthy controls (0/31). A combination of cerebrospinal fluid, white matter, and global signals reduced physiological artifacts (29). The DARTEL tool was used to compute transformations from individual native space to Montreal Neurological Institute space of 3 × 3 × 3 mm^3^. Then, we calculated the fALFF. A fast Fourier transform was used to transform the filtered time series of each voxel into a frequency domain and subsequently into a power spectrum. In each voxel, the square root of the signal was measured over 0.01–0.08 Hz. The fALFF was normalized through transformation to a z score. Finally, a 4-mm full-width, half-maximum Gaussian kernel was used for spatial smoothing.

### Statistic

Imaging differences between obesity and HC were measured using a two-sample *t*-test and *p* < 0.001 for voxels, *p* < 0.05 for clusters, GRF corrected. The statistical analysis and plotting were based on Jamovi v2.2.2 (https://www.jamovi.org/) and GraphPad v 9.0 (https://www.graphpad.com/). The summaries of quantitative variables with a normal distribution are expressed as the means and standard deviations; categorical data are expressed as rates. The group comparisons between obese and healthy controls were performed by independent *t*-tests (the comparison of the gender distribution in each group was verified by a χ^2^ test). Then, we used the Pearson correlation analysis to detect the correlation between brain activity and impulsiveness. For an original significance of α = 0.05, we used the Bonferroni correction for multiple testing (α' = α/m, where m is the number of comparisons; in this study, m = 9).

We used a mediation test to explore the mediating effect of spontaneous brain activity on the relationship between impulsiveness personality and obesity. We used WebPower (http://psychstat.org/mediation) to calculate the sample size (path a = −0.5, path b = −0.5, power = 0.8) for this mediation analysis. We used the general linear model mediation model (jamm Advanced Mediation Models v1.0.5) in Jamovi to perform the mediation analysis. A normal bootstrap method was used to construct a 95% confidence interval for significance testing of the mediating effects. The mediation analysis was to test the hypothesis that spontaneous brain activity mediated the relationship between impulsiveness and BMI. We also checked the reverse model, that spontaneous brain activity mediates BMI, which influences non-planning impulsiveness (see Supplementary Material). Following the mediation test, we also performed hierarchical clustering based on the significant brain activity and impulsiveness to explore how the impulsiveness and brain activity will be the intermediate phenotype for obesity. And after that, the χ^2^ test for association was used to test the association between brain activity and impulsiveness. The number of participants finally included in all data was 35+31, a total of 66.

## Results

### Group Comparison of fALFF

**Figure 1:**
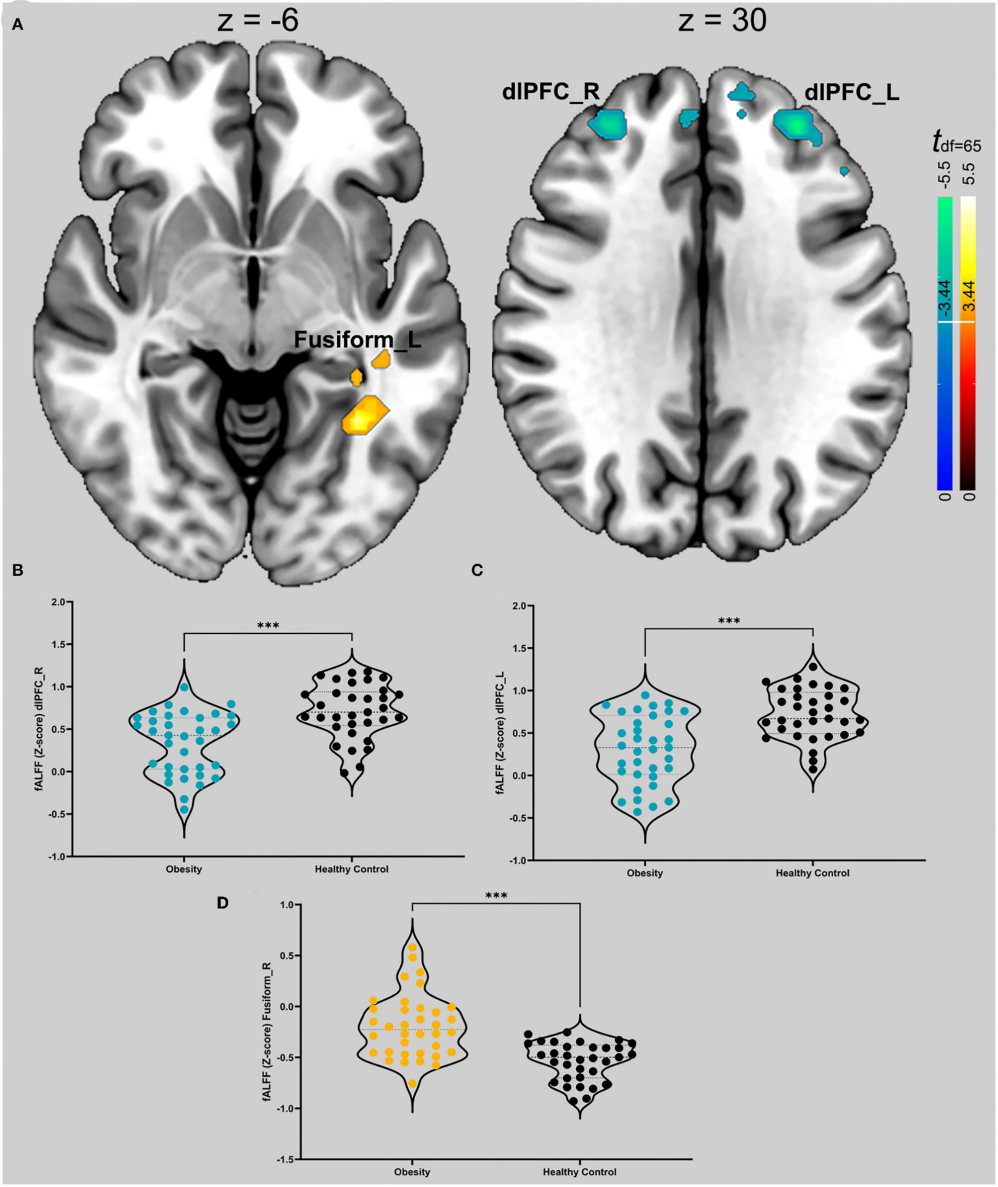
**(A)** t map of differences in spontaneous activity of the groups. Hot colors (red to bright yellow) indicate that obese patients have higher spontaneous brain activity in those regions; cool colors (blue) indicate that obese patients have lower spontaneous activity. The thresholds for the two comparisons are ±3.44. The slice on the graph is the cross section of the vertical axis with the maximum threshold. **(B)** Violin diagrams of the intensity values corresponding to the dlPFC of the two groups on the right. The colored dots in the figure are obese patients, and the black dots are healthy controls. The dotted lines represent the first, second, and third quantiles. We used an independent samples t test. *** Indicates *p* < 0.001 in **(A–D)**. **(C)** Violin graphs of the intensity values corresponding to the left dlPFC in the two groups. **(D)** Violin graphs of the intensity values corresponding to the left fusiform gyrus in the two groups.

We found that patients with obesity had a lower fALFF in the bilateral dlPFC (x = −30, y = 45, z = 30, k = 122, max-*t* = −5.62; x = 30, y = 45, z = 30, max-*t* = −4.91, k = 95) and a higher fALFF in the left fusiform region (x = −33, y = −51, z = −6, max-*t* = 5.15, k = 54). More detail is shown in Figure 1.

### Correlation Between fALFF and Impulsiveness

**Figure 2:**
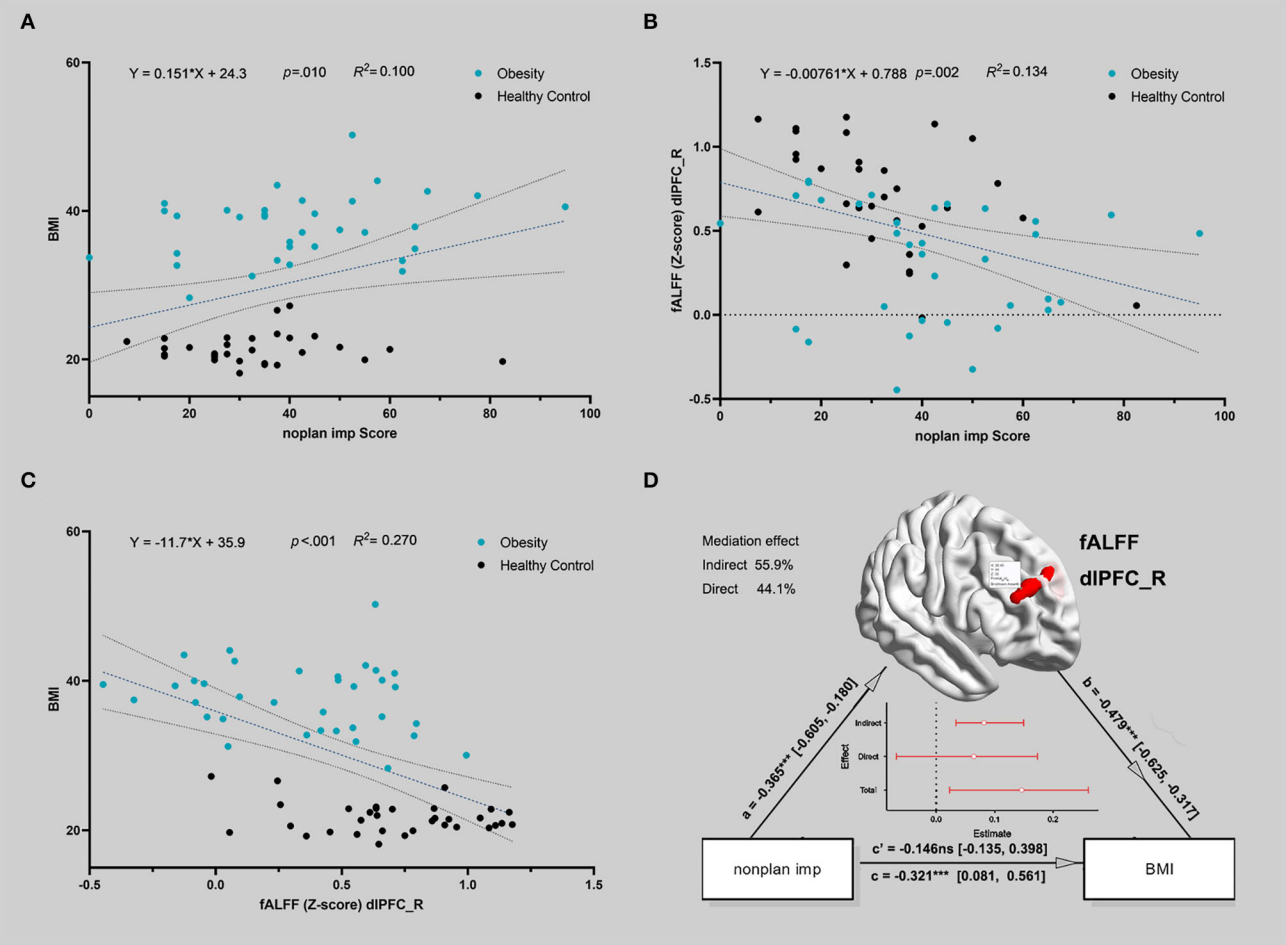
The correlation between the activity of the brain region during delayed gratification and the severity of IGD. **(A)** Non-planning impulsiveness and body mass index (BMI) were positively correlated in all participants. The blue dots represent patients, and the black dots represent healthy controls. A linear fit was conducted, and the dotted line indicates the 95% confidence interval of the dotted line (in **A–C**). **(B)** Non-planning impulsiveness and the fALFF in the right dlPFC were negatively correlated in all participants. **(C)** The fALFF in the right dlPFC and BMI were negatively correlated in all participants. **(D)** The indirect path (*via* lower fALFF in the right dlPFC) completely mediated the relationship between non-planning impulsiveness and BMI for all participants. The red line in the figure represents the standardized estimation coefficient; the y-intercept indicates that spontaneous brain activity mediates the influence of impulsivity on obesity.

The Pearson correlation analysis showed that non-planning impulsiveness was positively correlated with BMI (df = 65, r = 0.34, *p* = 0.005) and negatively correlated with the fALFF in the right dlPFC (df = 65, r = −0.38, *p* = 0.002); the fALFF in the right dlPFC was negatively correlated with BMI (df = 65, r = −0.52, *p* < 0.001) (Figures 2A–C).

### Mediation Results

**Figure 3:**
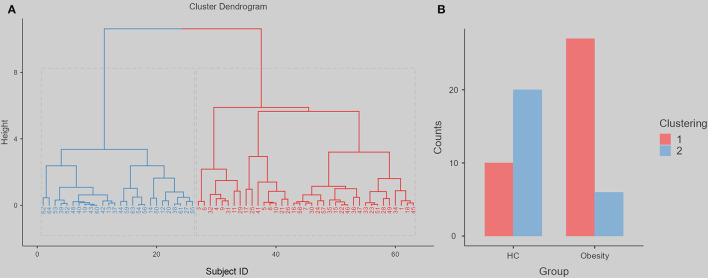
The hierarchical clustering dendrogram and following difference between obesity group and HC. We use the height 8, can divide all the participants into two clustering. The red represents clustering 1, the blue represents clustering 2. **(A)** The Cluster Dendrogram. **(B)** The Group Counts for each clustering.

Figure 2D shows that the indirect path *via* a lower fALFF (a^*^b, beta = 0.175, standard error = 0.065, 95% confidence interval = [0.075, 0.329], Z = 2.697, *p* = 0.007, mediation = 54.48%) completely mediated the direct path from non-planning impulsiveness to BMI (c, beta = 0.146, standard error = 0.133, 95% confidence interval = [−0.135, 0.398], Z = 1.098, *p* = 0.272, mediation = 45.52%). Additionally, the reverse model was estimated, and its results did not support the opposite relationship, that spontaneous brain activity mediated BMI and led to more non-planning impulsiveness. This model is discussed in more detail in the Supplementary Material. The χ^2^ test on the hierarchical clustering (including the fALFF in right dlPFC and non-planning impulsiveness score) also showed a higher rate of Cluster 1 in the obese group than in the HC group (χ2 value = 15.241, *p* < 0.001) (Figure 3).

## Discussion

This study aimed to explore whether brain activity can mediate the relationship between individual impulsiveness and obesity. Our analysis yielded two main results. This study was the first to find that obese patients have significantly lower spontaneous brain activity in the dlPFC, which supports our first hypothesis. Second, we found that the fALFF in the right dlPFC completely mediated the relationship between non-planning impulsiveness and obesity. These results indicate that spontaneous resting brain activity is a potential biomarker for obesity-related impulsiveness and regulates obesity outcomes.

Resting-state fMRI seems to be more strongly correlated with impulsive personality traits than obesity. First, not all the trait impulsiveness subscales correlate with obesity (30). In our study, we found that patients with obesity had increased non-planning impulsiveness than that of HCs. The results we obtained are not entirely consistent with previous results. The majority of previous surveys of obese patients showed higher impulsivity to exercise or attention than healthy individuals (25). Only a few studies related to food addiction have reported that unplanned impulsivity predicts BMI (31), which may indicate a significant number of potential food addicts in obese people. Further unplanned impulses also modulate the efficacy of transcranial direct current stimulation as an appetite intervention in obese patients (32). The relationship between non-planning impulsiveness and loss of control during a Binge maybe indicate the underlaying brain function altered in obesity (33). We found a significant decrease in the fALFF in the bilateral dlPFC in patients with obesity, which may indicate that the fALFF in the dlPFC is a relatively stable potential biomarker for obesity. Previous studies have reported many changes in brain regions in obese patients, such as decreased ALFF values in the hippocampus and putamen after bariatric surgery (20). Our findings contribute new evidence about cortical activity in obese patients. A previous meta-analysis showed that changes in the dlPFC are common in patients with obesity and patients with substance use disorders (34). This may be because obese patients undergo outpatient bariatric and metabolic surgery and therefore have more difficulty reducing food cravings, similar to a food addiction (35).

The dlPFC results may provide new insights into the treatment of obesity. This region is involved in important cognitive functions such as attention, memory, and executive control (36). The dlPFC is an emerging target area for non-invasive brain stimulation psychiatric interventions, such as treating major depression disorder, obsessive-compulsive disorder, and addiction (37). The dlPFC participates in several functional-based brain networks (38), such as the salience network (SN) and executive control network (ECN), which are related to impulsiveness (39). The SN is the key network for labeling relevant information. ECN controls reward-based decision-making. As previous studies have pointed out, coordinated alterations in the SN, ECN, and default mode network (DMN) facilitate the diagnosis and treatment of obesity (40). The clustering analysis based on fALFF in right dlPFC and non-planning impulsiveness score showed a higher rate of Cluster 1 in the obesity group than in the HC group. Non-planning impulsivity in the obesity population is more of an intermediate phenotype, and spontaneous neural activity in the right dlPFC may be a potential biomarker.

Most intriguingly, spontaneous brain activity can explain the impact of impulsivity on obesity outcomes, and its effect is very strong. Previous studies suggest that the impaired impulse control associated with obesity is caused by imbalanced dynamic cross-network interactions (41). There are many treatments for obesity, but the high levels of impulsiveness, anxiety, binge eating, food addiction, and other mental illness symptoms associated with obesity have made it unrealistic to develop treatments for weight loss (42). Even the most effective bariatric surgery is still associated with the risk of regaining weight, and psychotic symptoms are an important predictor of this weight gain. Furthermore, hierarchical clustering supports the use of fALFF to study obesity, and the involvement of unplanned impulses suggests that it is no longer appropriate to treat obesity as a mere metabolic disorder problem (43). There is an urgent need to develop effective, safe, and easy-to-use obesity treatment options such as non-invasive brain stimulation. However, the mediation effect is simply a statistical result; thus, we must be careful when concluding, such as theories that interventions addressing food-related impulsivity are more effective than other interventions (44). Several studies have investigated the interventional effect of non-inclination brain stimulation in obese patients. Early rTMS intervention in the left dlPFC of 29 obese patients effectively reduced fat mass and appetite (45). Recently, deep coils were used to stimulate the bilateral insula and prefrontal cortex of 9 patients, which was found to effectively reduce body weight and found an increase in prefrontal functional connectivity (46). This may suggest that non-invasive brain stimulation may be an effective treatment option for obesity, a multifactorial confounding disease. In addition, the relatively weaker but more portable transcranial direct current stimulation may have wider applicability. For example, a combined hypocaloric diet may have been effective in reducing sweets cravings in overweight individuals (47), and changes in neural activity may themselves have potential roles as predictive biomarkers for tDCS and cognitive training (CT) in the dlPFC The resulting prefrontal EEG asymmetry predicts a reduction in BMI (48). To improve treatment outcomes, more studies need to determine pre-treatment biomarkers of treatment outcomes to determine which patients may respond to specific treatments.

This study has several limitations. First, our patients were recruited mainly from a population in good health and with high socioeconomic status. This may introduce bias in our research because the sample may be more concerned with the quality of life and have a higher level of social support than other samples. Follow-up research should use more random sampling methods. Second, cross-sectional comparisons cannot make proper causal inferences. This study did not include anxiety and depressive mood in our analysis, and we believe that this comorbidity factor would weaken the overall statistical power if controlled for as a covariate. Especially when anxiety and depression themselves are highly correlated with obesity (49), it is a very dangerous move to try to eliminate the major factors, which may be the abnormal phenotypes shared by anxiety and depression with obesity. As our previous study explored (50). Therefore, future exploratory research should use longitudinal methods. Third, our participants were mainly female; thus, gender differences in impulsivity and obesity must be considered (51). Obesity is the last observable result, resulting from excess consumption and intake of calories. We hypothesize that obese people are less physically active than healthy people under modern living conditions (52). Due to the lack of monitoring of subject exercise levels in this study, we were not able to discuss obesity outcomes within a unified dynamic energy balance framework (53). As part of future studies, we will also include a self-reported International Physical Activity Questionnaire or exercise monitoring wristbands for control measurements. Add more evidence regarding “healthier” community building designs as a means of reducing obesity (54).

## Conclusion

In conclusion, these results indicate that the dlPFC can serve as a biomarker of obesity and that its activity mediates the path from increased non-planning impulsiveness to higher BMI. Our results may facilitate the development of novel treatment strategies, such as non-invasive brain stimulation, and advance the personalized treatment of obesity.

## Data Availability Statement

The original contributions presented in the study are included in the article/Supplementary Material further inquiries can be directed to the corresponding author/s.

## Ethics Statement

The studies involving human participants were reviewed and approved by the human research review board of Shanghai Jiao Tong University Affiliated Sixth People's Hospital. The patients/participants provided their written informed consent to participate in this study. Written informed consent was obtained from the individual(s) for the publication of any potentially identifiable images or data included in this article.

## Author Contributions

X-DH, Z-QL, and H-WZ: writing-original draft. TX, QL, and LL: resources. H-TC: writing-review and editing. HZ: project administration, methodology, and software. TX: resources and conceptualization. T-FY: conceptualization and supervision. All authors contributed to the article and approved the submitted version.

## Funding

This work was supported by clinical retrospective study of Shanghai Jiao Tong University Affiliated Sixth People's Hospital (YNHG201912 to H-WZ), the Natural Science Foundation of China (82171486), Natural Science Foundation of Shanghai (21ZR1448400 to TX), the Interdisciplinary Program of Shanghai Jiao Tong University (YG2021ZD23 to TX), General Science Foundation of Shanghai Sixth People's Hospital (YNMS202114 to TX).

## Conflict of Interest

QL was employed in Siemens Healthcare Ltd. The remaining authors declare that the research was conducted in the absence of any commercial or financial relationships that could be construed as a potential conflict of interest.

## Publisher's Note

All claims expressed in this article are solely those of the authors and do not necessarily represent those of their affiliated organizations, or those of the publisher, the editors and the reviewers. Any product that may be evaluated in this article, or claim that may be made by its manufacturer, is not guaranteed or endorsed by the publisher.
