# Supplementary material for: How Impulsiveness Influences Obesity: The Mediating Effect of Resting-State Brain Activity in the dlPFC
Source: Front Psychiatry. 2022 May 10;13:873953. doi: 10.3389/fpsyt.2022.873953 (PMC9127259; doi:10.3389/fpsyt.2022.873953)
Supplement: Supplementary file 1 [file Data_Sheet_1.docx]

Supplement Note

**Methods**

Details of multiple corrections in fMRI static and correlation analysis. Initially, the comparison of resting-state fMRI requires multiple corrections. Global signals are widely used as regressors or normalization factors to remove the effects of global variations (Liu et al., 2017). Thus, we compare two conditions: 1) use global signal as covariable and remove these effects, 2) do not care about the global signal, in the main manuscript, we report results that do not care about the global signal, in this supplement, we can see that even after removing the global signal, fMRI results remain robust.

Second, the multiple correction methods also have an effect on the results. Gaussian Random Field (GRF) was used to correct our results. As suggested in the previous study, we set single voxel p < .001, cluster level p < .05, two tails (Chen et al., 2018). For each condition, we must estimate smoothness on the statistical image directly (following FSL estimation). Then we can determine FWHM in each axis (x, y, z) and DLH. Voxel Z threshold for voxel p threshold 0.001 is: 3.290527. Calculate the minimum cluster size for voxel p threshold 0.001 and then cluster p threshold 0.05. Lastly, the cluster size threshold will be applied to the original t image. In this study, the threshold number for condition 1 is 63 and the T value is 3.437194, while the size for condition 2 is 54 and the T value is 3.441749.

Following the fMRI comparison, we found three significant clusters in the fALFF comparison. The BIS has three subscales. A Bonferroni correction is applied. (Jafari & Ansari-Pour, 2019). Our registration in OSF enables us to calculate all the possible correlations between brain altered and BIS subscale, thus ensuring that each analysis is accurate. There are 9 possible statistical tests for each *p*, so divide each *p* by 3*3.

Lastly, the mediation model faces a serious question about how to choose the dependent variable, mediator and predictor. Our hypothesis is that the brain mediates impulsivity and BMI. According to the theory, obesity is caused by impaired self-control (Teixeira et al., 2015). However, we are doing a cross-sectional study, and we still need to test the opposite direction. Is impulse a consequence of obesity?

**Results**

**fMRI comparison after regress the global signals**

Add global signals as covariates to analysis the fALFF. The results showed that, compare with healthy controls, patients with obesity have higher fALFF value in the left fusiform (x=-30, y=45, z=30, k =179, max-t=-5.19; x=30, y=45, z=30, max-t =-5.88,k=190, BA 10), and lower fALFF value in the bilateral dlPFC (x=-33, y=-51, z=-6, max-*t*=5.45, k=87, BA19).


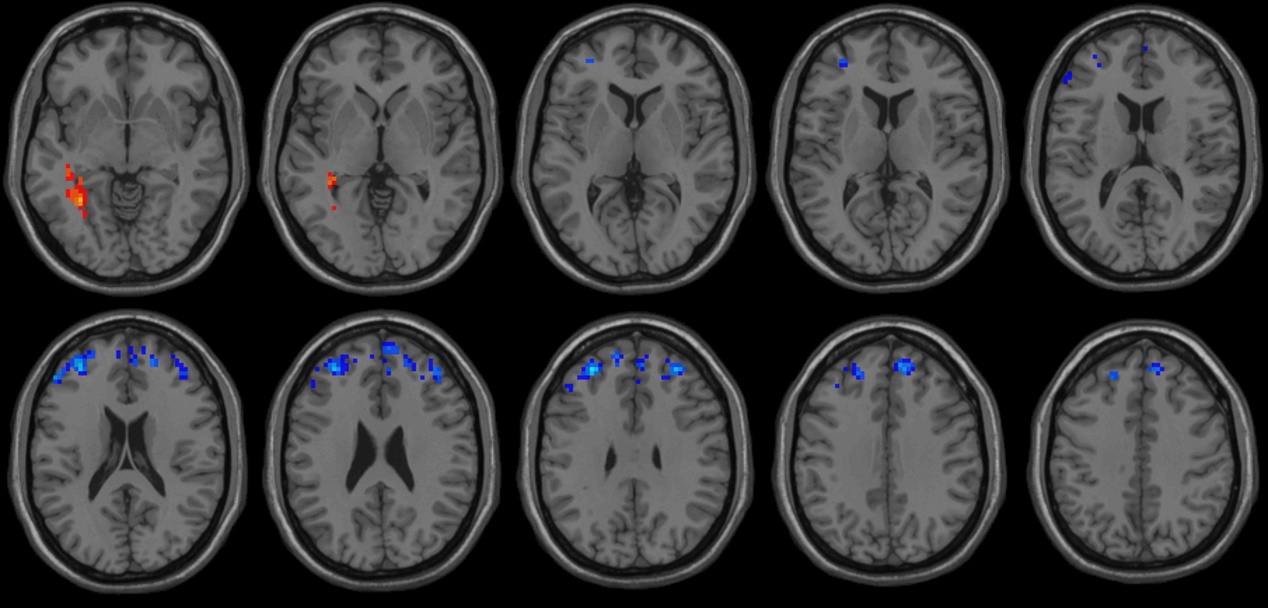


**SFigure 1 the group comparison between Obesity and Healthy Control with global**

signal remove. The position for each frame [z= -5 0 5 10 15; 20 25 30 35 40]. Red to bright yellow indicate that the fALFF value of obese patients is higher, and dark blue to light blue indicate that the fALFF value of obese patients is lower.

**The correlation between brain activity and BIS subscale**

The correlations analyses showed that after multiple corrections the right dlPFC, nonplan imp and BMI is the only path survival.

**STable 1 The correlation between brain activity and BIS subscale**


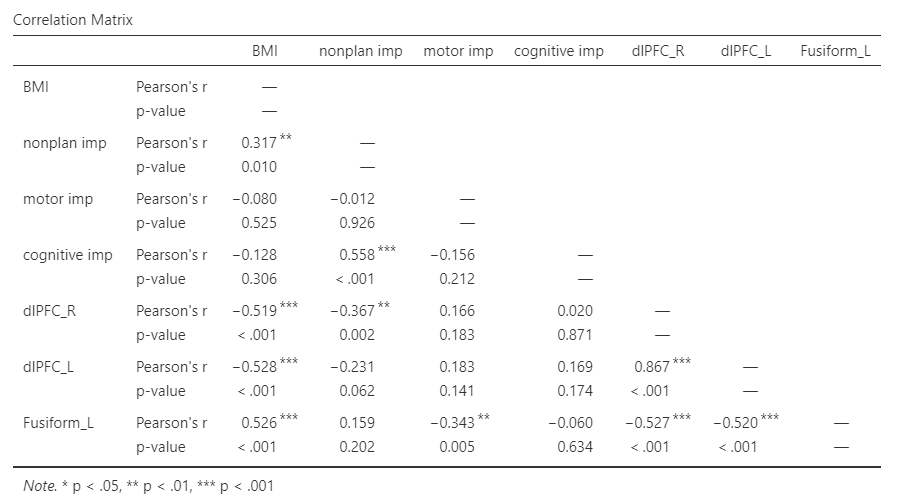


**The model from BMI**

The FigS2 showed that the mediation model from BMI → brain activation → non-planning impulsiveness estimates. The results showed that the indirect and direct pathway have cross zero. So, this model is not support by our evidence. More details showed in the TableS2 and TableS3.

**STable 2 Mediation Estimates from nonplanning impulsivity to BMI**

|  | | | | | | | | | | **95% Confidence Interval** | | | |  | | | | | |
| --- | --- | --- | --- | --- | --- | --- | --- | --- | --- | --- | --- | --- | --- | --- | --- | --- | --- | --- | --- |
| **Effect** | | **Label** | | | **Estimate** | | | **SE** | | **Lower** | | **Upper** | | **Z** | | **p** | | **% Mediation** | |
| Indirect |  | | a × b |  | | 0.143 |  | 0.080 |  | -0.009 |  | 0.311 |  | 1.788 |  | 0.074 |  | 45.723 |  |
| Direct |  | | c |  | | 0.170 |  | 0.157 |  | -0.137 |  | 0.500 |  | 1.079 |  | 0.281 |  | 54.277 |  |
| Total |  | | c + a × b |  | | 0.312 |  | 0.120 |  | 0.082 |  | 0.554 |  | 2.597 |  | 0.009 |  | 100.000 |  |
|  | | | | | | | | | | | | | | | | | | | |

**STable 3 Path Estimates from nonplanning impulsivity to BM**

|  | | | | | | | | | | | | | | | | | | | | | | | |
| --- | --- | --- | --- | --- | --- | --- | --- | --- | --- | --- | --- | --- | --- | --- | --- | --- | --- | --- | --- | --- | --- | --- | --- |
|  | | | | | | | | | | | | **95% Confidence Interval** | | | | | |  | | | | | |
|  | |  | |  | | **Label** | | **Estimate** | | **SE** | | **Lower** | | | **Upper** | | | **Z** | | | **p** | | |
| zBMI |  | → |  | zdlfpc |  | a |  | -0.514 |  | 0.110 |  | -0.744 |  | -0.303 | |  | -4.661 | |  | < .001 | |  |  |
| zdlfpc |  | → |  | zNoplanImp |  | b |  | -0.278 |  | 0.149 |  | -0.566 |  | 0.014 | |  | -1.866 | |  | 0.062 | |  |  |
| zBMI |  | → |  | zNoplanImp |  | c |  | 0.170 |  | 0.157 |  | -0.137 |  | 0.500 | |  | 1.079 | |  | 0.281 | |  |  |
|  | | | | | | | | | | | | | | | | | | | | | | | |

 
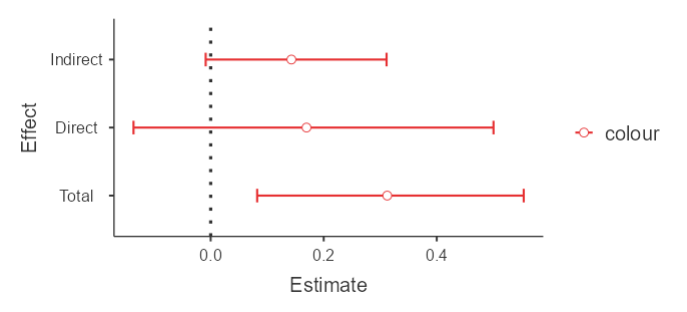


**SFigure 2 The estimate plot for mediation model from BMI → brain activation → non-planning impulsiveness. The red horizontal line shows whether the 95% confidence interval passes through the vertical dotted line, which means that the effect size is unstable.**

**STable 4 correlation between Obeisty onset time and other factors**

|  | |  | | **FatTime** | |
| --- | --- | --- | --- | --- | --- |
| FatTime |  | Spearman's rho |  | — |  |
|  |  | p-value |  | — |  |
|  |  | N |  | — |  |
| BMI |  | Spearman's rho |  | -0.302 |  |
|  |  | p-value |  | 0.083 |  |
|  |  | N |  | 34 |  |
| dlPFC_L |  | Spearman's rho |  | -0.001 |  |
|  |  | p-value |  | 0.995 |  |
|  |  | N |  | 34 |  |
| dlPFC_R |  | Spearman's rho |  | 0.167 |  |
|  |  | p-value |  | 0.345 |  |
|  |  | N |  | 34 |  |
| nonplan imp |  | Spearman's rho |  | -0.052 |  |
|  |  | p-value |  | 0.769 |  |
|  |  | N |  | 34 |  |
| Fusiform_L |  | Spearman's rho |  | 0.098 |  |
|  |  | p-value |  | 0.580 |  |
|  |  | N |  | 34 |  |
| motor imp |  | Spearman's rho |  | 0.053 |  |
|  |  | p-value |  | 0.767 |  |
|  |  | N |  | 34 |  |
| cognitive imp |  | Spearman's rho |  | 0.176 |  |
|  |  | p-value |  | 0.319 |  |
|  |  | N |  | 34 |  |
| education (years) |  | Spearman's rho |  | -0.009 |  |
|  |  | p-value |  | 0.958 |  |
|  |  | N |  | 34 |  |
| average imp |  | Spearman's rho |  | 0.014 |  |
|  |  | p-value |  | 0.935 |  |
|  |  | N |  | 34 |  |

**STable 5 The OLS general linear model predict BMI by resting state right dlPFC fALFF and nonplanning impulsivity**

|  | | | |
| --- | --- | --- | --- |
|  |  |  |  |
| **Model Info** | |  | |
| Estimate |  | Linear model fit by OLS |  |
| Call |  | BMI ~ 1 + dlPFC_R + `nonplan imp` + dlPFC_R:`nonplan imp` |  |
| R-squared |  | 0.324 |  |
| Adj. R-squared |  | 0.291 |  |
|  | | | |

**STable 6 The ANOVA analysis of each predictors**

|  | | | | | | | | | | | |
| --- | --- | --- | --- | --- | --- | --- | --- | --- | --- | --- | --- |
|  |  |  |  |  |  |  |  |  |  |  |  |
| **ANOVA Omnibus tests** | | **SS** | | **df** | | **F** | | **p** | | **η²p** | |
| Model |  | 467.948 |  | 3 |  | 8.735 |  | < .001 |  | 0.297 |  |
| nonplan imp |  | 100.762 |  | 1 |  | 1.725 |  | 0.194 |  | 0.125 |  |
| dlPFC_R |  | 340.632 |  | 1 |  | 5.830 |  | 0.019 |  | 0.214 |  |
| nonplan imp ✻ dlPFC_R |  | 26.554 |  | 1 |  | 0.454 |  | 0.503 |  | 0.007 |  |
| Residuals |  | 3622.432 |  | 62 |  |  |  |  |  |  |  |
| Total |  | 4090.380 |  | 65 |  |  |  |  |  |  |  |
|  | | | | | | | | | | | |


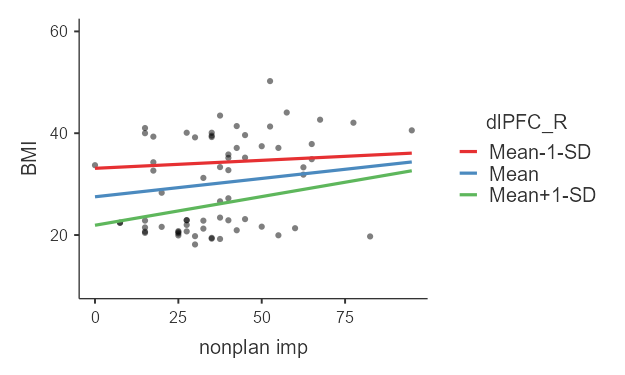


SFigure 3 The simple effect of dlPFC_R fALFF between nonplanning impulsiveness and BMI

STable 7 **The t test compares male and female participants in main factors**

| Welch's t | | **Statistic** | | | **df** | | ***p*** | | Cohen's d | | |  |
| --- | --- | --- | --- | --- | --- | --- | --- | --- | --- | --- | --- | --- |
| BMI |  |  | 0.437 |  | | 24.182 |  | 0.666 |  | 0.126 |  | |
| dlPFC_L |  |  | 0.542 |  | | 21.131 |  | 0.593 |  | 0.164 |  | |
| dlPFC_R |  |  | -0.153 |  | | 20.182 |  | 0.880 |  | -0.047 |  | |
| Fusiform_L |  |  | 0.088 |  | | 25.948 |  | 0.931 |  | 0.025 |  | |
| age |  |  | -0.719 |  | | 20.125 |  | 0.481 |  | -0.222 |  | |
| education (years) |  |  | -1.662 |  | | 30.554 |  | 0.107 |  | -0.453 |  | |
| education level |  |  | -1.412 |  | | 29.587 |  | 0.168 |  | -0.388 |  | |
| nonplan imp |  |  | 1.305 |  | | 23.560 |  | 0.204 |  | 0.384 |  | |
| znonplan imp |  |  | 1.305 |  | | 23.560 |  | 0.204 |  | 0.384 |  | |
| motor imp |  |  | 1.396 |  | | 23.047 |  | 0.176 |  | 0.413 |  | |
| cognitive imp |  |  | 0.138 |  | | 20.313 |  | 0.892 |  | 0.043 |  | |
| imp_total |  |  | 1.422 |  | | 22.885 |  | 0.168 |  | 0.422 |  | |

STable 8 Homogeneity of Variances Tests

|  | | | | | | | | | | | |
| --- | --- | --- | --- | --- | --- | --- | --- | --- | --- | --- | --- |
|  |  |  |  |  |  |  |  |  |  |  |  |
|  | |  | | **F** | | **df** | | **df2** | | **p** | |
| BMI |  | Levene's |  | 0.396 |  | 1 |  | 66 |  | 0.531 |  |
|  | | Variance ratio |  | 0.925 |  | 51 |  | 15 |  | 0.793 |  |
| dlPFC_L |  | Levene's |  | 1.804 |  | 1 |  | 66 |  | 0.184 |  |
|  | | Variance ratio |  | 0.629 |  | 51 |  | 15 |  | 0.219 |  |
| dlPFC_R |  | Levene's |  | 4.135 |  | 1 |  | 66 |  | 0.046 |  |
|  | | Variance ratio |  | 0.535 |  | 51 |  | 15 |  | 0.099 |  |
| Fusiform_L |  | Levene's |  | 0.002 |  | 1 |  | 66 |  | 0.965 |  |
|  | | Variance ratio |  | 1.095 |  | 51 |  | 15 |  | 0.889 |  |
| age |  | Levene's |  | 5.239 |  | 1 |  | 66 |  | 0.025 |  |
|  | | Variance ratio |  | 0.529 |  | 51 |  | 15 |  | 0.094 |  |
| education (years) |  | Levene's |  | 1.973 |  | 1 |  | 64 |  | 0.165 |  |
|  | | Variance ratio |  | 1.487 |  | 49 |  | 15 |  | 0.404 |  |
| education level |  | Levene's |  | 0.016 |  | 1 |  | 64 |  | 0.899 |  |
|  | | Variance ratio |  | 1.396 |  | 49 |  | 15 |  | 0.488 |  |
| nonplan imp |  | Levene's |  | 0.087 |  | 1 |  | 64 |  | 0.768 |  |
|  | | Variance ratio |  | 0.834 |  | 49 |  | 15 |  | 0.608 |  |
| motor imp |  | Levene's |  | 0.411 |  | 1 |  | 64 |  | 0.524 |  |
|  | | Variance ratio |  | 0.786 |  | 49 |  | 15 |  | 0.510 |  |
| cognitive imp |  | Levene's |  | 1.677 |  | 1 |  | 64 |  | 0.200 |  |
|  | | Variance ratio |  | 0.527 |  | 49 |  | 15 |  | 0.094 |  |
| imp_total |  | Levene's |  | 0.829 |  | 1 |  | 64 |  | 0.366 |  |
|  | | Variance ratio |  | 0.771 |  | 49 |  | 15 |  | 0.480 |  |
| Note. Additional results provided by *moretests* | | | | | | | | | | | |
|  | | | | | | | | | | | |

STable 9 Tests of Normality

|  | |  | | **statistic** | | **p** | |
| --- | --- | --- | --- | --- | --- | --- | --- |
| BMI |  | Shapiro-Wilk |  | 0.897 |  | < .001 |  |
|  | | Kolmogorov-Smirnov |  | 0.197 |  | 0.009 |  |
|  | | Anderson-Darling |  | 2.807 |  | < .001 |  |
| dlPFC_L |  | Shapiro-Wilk |  | 0.972 |  | 0.121 |  |
|  | | Kolmogorov-Smirnov |  | 0.079 |  | 0.757 |  |
|  | | Anderson-Darling |  | 0.552 |  | 0.149 |  |
| dlPFC_R |  | Shapiro-Wilk |  | 0.965 |  | 0.054 |  |
|  | | Kolmogorov-Smirnov |  | 0.105 |  | 0.410 |  |
|  | | Anderson-Darling |  | 0.779 |  | 0.041 |  |
| Fusiform_L |  | Shapiro-Wilk |  | 0.954 |  | 0.013 |  |
|  | | Kolmogorov-Smirnov |  | 0.110 |  | 0.356 |  |
|  | | Anderson-Darling |  | 0.949 |  | 0.015 |  |
| age |  | Shapiro-Wilk |  | 0.842 |  | < .001 |  |
|  | | Kolmogorov-Smirnov |  | 0.139 |  | 0.132 |  |
|  | | Anderson-Darling |  | 2.423 |  | < .001 |  |
| education (years) |  | Shapiro-Wilk |  | 0.937 |  | 0.002 |  |
|  | | Kolmogorov-Smirnov |  | 0.162 |  | 0.062 |  |
|  | | Anderson-Darling |  | 1.673 |  | < .001 |  |
| education level |  | Shapiro-Wilk |  | 0.886 |  | < .001 |  |
|  | | Kolmogorov-Smirnov |  | 0.207 |  | 0.007 |  |
|  | | Anderson-Darling |  | 2.971 |  | < .001 |  |
| nonplan imp |  | Shapiro-Wilk |  | 0.961 |  | 0.037 |  |
|  | | Kolmogorov-Smirnov |  | 0.115 |  | 0.351 |  |
|  | | Anderson-Darling |  | 0.815 |  | 0.033 |  |
| motor imp |  | Shapiro-Wilk |  | 0.943 |  | 0.005 |  |
|  | | Kolmogorov-Smirnov |  | 0.154 |  | 0.087 |  |
|  | | Anderson-Darling |  | 1.380 |  | 0.001 |  |
| cognitive imp |  | Shapiro-Wilk |  | 0.948 |  | 0.008 |  |
|  | | Kolmogorov-Smirnov |  | 0.118 |  | 0.320 |  |
|  | | Anderson-Darling |  | 1.191 |  | 0.004 |  |
| imp_total |  | Shapiro-Wilk |  | 0.958 |  | 0.025 |  |
|  | | Kolmogorov-Smirnov |  | 0.115 |  | 0.349 |  |
|  | | Anderson-Darling |  | 1.014 |  | 0.011 |  |
| Note. Additional results provided by *moretests* | | | | | | | |
|  | | | | | | | |

Bibliography

Chen, X., Lu, B., & Yan, C.-G. (2018). Reproducibility of R-fMRI metrics on the impact of different strategies for multiple comparison correction and sample sizes. *Human Brain Mapping*, *39*(1), 300–318. https://doi.org/10.1002/hbm.23843

Jafari, M., & Ansari-Pour, N. (2019). Why, when and how to adjust your P values? *Cell journal*, *20*(4), 604–607. https://doi.org/10.22074/cellj.2019.5992

Liu, T. T., Nalci, A., & Falahpour, M. (2017). The global signal in fMRI: Nuisance or Information? *Neuroimage*, *150*, 213–229. https://doi.org/10.1016/j.neuroimage.2017.02.036

Teixeira, P. J., Carraça, E. V., Marques, M. M., Rutter, H., Oppert, J.-M., De Bourdeaudhuij, I., Lakerveld, J., & Brug, J. (2015). Successful behavior change in obesity interventions in adults: a systematic review of self-regulation mediators. *BMC Medicine*, *13*, 84. https://doi.org/10.1186/s12916-015-0323-6
